# Ecological genomics beyond genome reports

**DOI:** 10.1093/g3journal/jkae309

**Published:** 2025-03-18

**Authors:** Koen J F Verhoeven, Tanja Pyhäjärvi

**Affiliations:** Department Terrestrial Ecology, Netherlands Institute of Ecology (NIOO-KNAW), Droevendaalsesteeg 10, 6708 PB Wageningen, The Netherlands; Department of Forest Sciences, University of Helsinki, Viikinkaari 1, 00790 Helsinki, Finland

## Abstract

With ongoing advances in sequencing and bioinformatics, the availability of reference genomes is spreading rapidly across the tree of life. Through genomics-enabled research, we are increasingly able to study the biology that we are interested in for the species that we care about. This expands the scope of our field. While research in traditional model organisms will continue to enable cumulative knowledge and breakthroughs in human biology and agriculture, genetic and genomic insights from a broader diversity of species can unlock useful and unique information on how organisms can deal with specific ecological and environmental challenges.

The fields of genetics and genomics have been built on model organisms, and those organisms remain critical to biological research today. By focusing on a single study species, labs can make efficient use of each other's resources, methodologies, and biological insights, generating new knowledge through cumulative effort. This approach is incredibly powerful and has brought us most of what we know about how genes affect traits and organisms. *G3* reflects this reality: studies on *Drosophila*, *Caenorhabditis elegans*, mouse, baker's yeast, and *Arabidopsis* have contributed almost one third of all empirical research articles in G3 in recent years. Yet, G3 also encourages genetic and genomic research beyond model organisms (McIntyre 2024), and the diversity of organisms represented by *Genome Reports* points to a new trajectory for our field.

Model organisms represent only a tiny sliver of the diversity of the tree of life, and the genomes and epigenomes of most species remain unexplored. The principle of homology allows us to gain some understanding of many aspects of these unexplored genomes based on knowledge gained from model species. Research in model organisms has greatly contributed to our understanding of human biology and to crop development; however, the many unexplored taxa also possess novel evolutionary solutions to specific ecological challenges that are not shared even with their most closely related model species. Evolutionary solutions in specific taxa have given us Taq polymerase (from the thermophilic hot springs bacteria *Thermus aquaticus*), GFP (green fluorescent protein, from the bioluminescent jellyfish *Aequorea victoria*), and countless drugs such as paclitaxel for chemotherapy, from Pacific yew, an evergreen conifer, to name just a few examples. Because the genomes and epigenomes of most species remain unexplored, many fascinating discoveries have yet to be made.

The sequencing revolution has changed the research landscape, and it's now realistic for many individual labs to sequence and assemble high quality genomes. We see this reflected in G3's Genome Reports. In 2024 G3 published more than 65 genome reports. There we read about genome assemblies of, for instance, stink bug (Shibata *et al*. 2024), the fungal parasite *Salmacisia buchloëana* (Benson *et al*. 2024), aquatic predatory Colorado pikeminnow (Mussmann 2024), several beetles (Ang *et al*. 2024; Arnqvist *et al*. 2024; Sylvester *et al*. 2024), slender anole (Pirani *et al*. 2024), multiple trees including black spruce (Lo *et al*. 2024), two culturally important *Vaccinium* berries (Albuja-Quintana *et al*. 2024; Hirabayashi *et al*. 2024), you name it! However, both the functional and structural annotation remains challenging, partly because annotation pipelines and models are built based on traditional model species characteristics (e.g., relatively small genome and gene sizes).

The publication of reference genomes from a widening group of species opens the door to a range of new research questions and opportunities. Reference-guided functional and comparative analysis can reveal genes or pathways that underpin evolutionary responses to the specific ecologies of these species (see for instance the analysis of resistance genes that are responsible for the large variation in resistance to rust disease that is observed in the threatened Whitebark pine (Neale *et al*. 2024)). Reference-guided genomic analysis also helps to reveal the genomic basis of local adaptation. Campbell and Hale (2024) evaluated the role of structural variants in Barramundi Perch and found indications that variation in adaptive life history traits between geographic lineages is linked to chromosomal inversions. As a further example, reference genomes also facilitate the analysis of expression and epigenetic regulation of the genome in species with unique life histories ad ecologies, such as duckweeds (Harkess *et al*. 2024).

Importantly, reference genomes enable the development of further resources for follow up genetic work. While novel technology is making real improvements in efficiency and cost effectiveness of research in model organisms (Blanchard *et al*. 2024), the impact of novel genomic tools is arguably larger for research in species outside traditional models. Interesting examples are the development of an amplicon panel for genotyping in Pacific oyster (Sutherland *et al*. 2024) and an efficient tool for gene editing in the Jewel wasp (Zhang *et al*. 2024). Such tools may open completely new research directions in these respective species.

The above examples from recent G3 issues show that such postreference genome follow-up research is starting to find its way to our journal. We encourage more submissions and are interested not only in publishing the genome reports per se, but also those manuscripts reporting the follow-up work and the unique genetic and genomic insights that can come from species with unique ecological and evolutionary characteristics. G3 values experimental and analytical rigor, and seeks to make available useful resources, over perceived impact. This philosophy fits well with studies in species that perhaps only a few labs in the world are working on—as often is the case for research on non-model organisms.

While model organisms are still critical for figuring out how genes affect traits and organisms, we are entering a research era in genetics and genomics where it is increasingly possible to unravel the biology that you are interested in. As the sequencing revolution and opportunities for experimental genetics spread over the tree of life, we anticipate fascinating discoveries. We are looking forward to seeing what you find now that you have a genome!
